# Zirkulärer Weichteildefekt nach prolongiertem Belassen eines strangulierenden Kompressionsstrumpfes

**DOI:** 10.1007/s00113-020-00951-y

**Published:** 2021-01-08

**Authors:** Sophia Juliane Mirtschink, Elisabeth Manke, Wolfgang Schneiders

**Affiliations:** Sektion Plastisch-Rekonstruktive Chirurgie, Elblandzentrum für Orthopädie und Unfallchirurgie, Elblandklinikum Riesa, Weinbergstraße 8, 01589 Riesa, Deutschland

**Keywords:** M.-peronaeus-brevis-Lappen, Gefäßgestielte Lappenplastik, Unterschenkelrekonstruktion, Weichteildefekt, Lokaler Lappen, Peroneus brevis muscle flap, Pedicled flap reconstruction, Lower limb reconstruction, Soft tissue defect, Local flap

## Abstract

Das prolongierte Belassen des Thrombosestrumpfes kann zu tiefgradigen Haut‑/Weichteildefekten führen. In dem konkreten Fall kam es zu einer zirkulären Nekrose mit freiliegender Sehne des M. tibialis anterior und der Achillessehne. Die Defektdeckung mittels M.-peronaeus-brevis-Lappen führte zu einer adäquaten Bedeckung der Achillessehne. Dieser Lappen stellt, insbesondere bei multimorbiden Patienten, aufgrund der kurzen Operations- und Narkosezeit eine gute Möglichkeit zur Defektdeckung am Unterschenkel dar. Dieser Fall sensibilisiert für die Wichtigkeit einer adäquaten Patientenschulung vor Beginn einer Kompressionstherapie.

## Abstract (en)

Wearing a compression stocking over a longer period of time can lead to deep skin and soft tissue defects. This article presents a case of a circular necrosis with an exposed tendon of the tibialis anterior muscle and the Achilles tendon. The use of a peroneus brevis muscle flap led to an adequate coverage of the exposed Achilles tendon. Due to the short operating and anesthesia times, this flap is a good option for lower leg reconstruction, particularly in multimorbid patients. This case raises awareness of the importance of adequate patient training before commencing compression therapy.

Defekte des Unterschenkels mit freiliegenden Sehnen oder Knochen stellen nach wie vor, v. a. je distaler sie lokalisiert sind, eine Herausforderung für jeden rekonstruktiv tätigen Chirurgen dar. Dabei fließen nicht nur die Defektlokalisation, -größe und -tiefe, sondern auch die Komorbiditäten des Patienten in den Entscheidungsprozess für die adäquate Lappendeckung ein. Für tiefgradige Defekte mittlerer Größe eignet sich, insbesondere auch bei multimorbiden Patienten, die distal gestielte Musculus-peronaeus-brevis-Lappenplastik.

In der vorliegenden Kasuistik wird ein in der internationalen Fachliteratur bisher noch nicht beschriebener Fall einer Strangulationsverletzung am Übergang vom mittleren zum distalen Unterschenkeldrittel durch einen zusammengerollten, über 3 Wochen belassenen Kompressionsstrumpf vorgestellt. Dies resultierte in einem zirkulären Defekt mit freiliegender Sehne des M. tibialis anterior und der Achillessehne.

## Falldarstellung

### Anamnese

Eine 69-jährigen Patientin stellte sich in Begleitung ihres betreuenden Neffen und der Polizei in unserer Notfallambulanz vor. Sie hatte etwa 3 Wochen einen zusammengerollten Thrombosestrumpf am linken Unterschenkel belassen, nachdem es ihr nicht gelang, diesen vollständig und eigenständig auszuziehen. Eine Vorstellung in der Notfallambulanz wurde längere Zeit von der Patientin abgelehnt. An Vorerkrankungen waren ein oral geführter Diabetes mellitus Typ 2, eine COPD sowie eine Zwangsstörung und ein Nikotinabusus bekannt.

### Klinischer und radiologischer Befund

**Figure 1:**
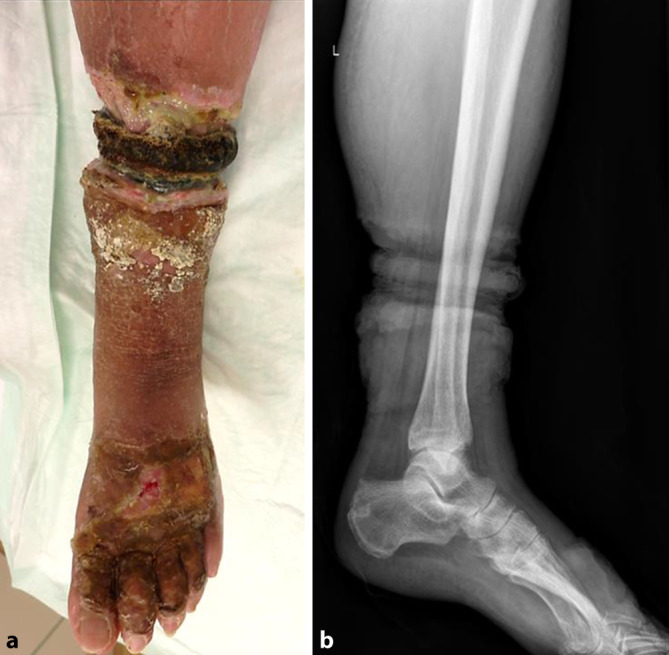

In der klinischen Untersuchung zeigte sich der Rand des Thrombosestrumpfes in die Weichteile eingewachsen (Abb. 1). Es imponierte ein faulig-jauchiger Geruch. Nach Aufweichen und Abziehen des Strumpfes bis zum eingerollten Gummirand ließen sich insbesondere interdigital sowie an Fußrücken und Fußsohle borkig-krustige Hautbeläge, teilweise mit oberflächlich blutenden Mazerationen nachweisen. Eine Rötung und Schwellung zogen sich bis unterhalb des Kniegelenks. Die Entzündungswerte waren mit einem CRP von 72 mg/l und Leukozyten von 10 Gpt/l initial erhöht. In der Röntgenuntersuchung des linken Unterschenkels und Fußes war eindrücklich die Weichteileinschnürung (Abb. 1) sichtbar.

### Therapie und Verlauf

**Figure 2:**
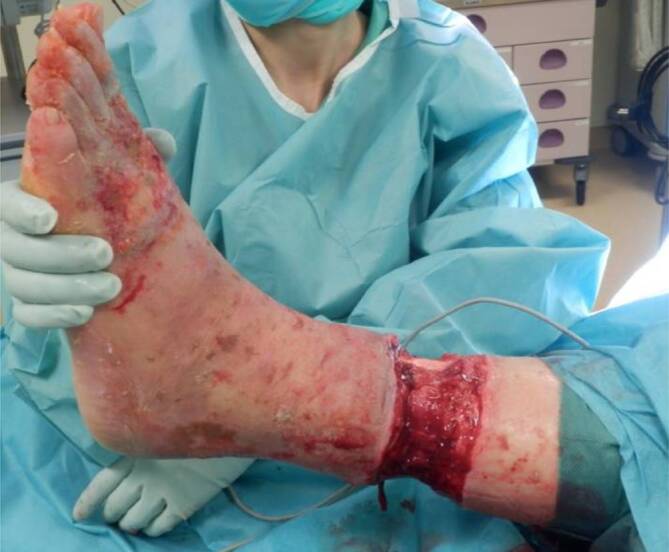

Am Aufnahmetag erfolgte operativ eine Entfernung des strangulierenden Thrombosestrumpfes. Es zeigte sich eine zirkuläre Nekrose mit freiliegender Sehne des M. tibialis anterior und der Achillessehne (Abb. 2). Die Defektgröße betrug 5,5 cm × 25 cm. Die Tibiavorderkante war durch eine dünne Schicht Weichteilgewebe bedeckt. Nach lokaler Antiseptikaeinwirkung wurde ein Vakuumverband angelegt. Eine Indikation zur Unterschenkelamputation bestand bei vitalem Fuß und palpatorisch vorhandenen peripheren Fußpulsen nicht.

In einer postoperativ durchgeführten CT-Angiographie zur Beurteilung der Durchblutungssituation stellten sich umschriebene „hard plaques“ in der A. poplitea mit ca. 50 % Durchmesserstenose, eine kräftige Kontrastierung des Truncus tibiofibularis sowie der A. tibialis anterior und fibularis beidseits dar. Die A. tibialis posterior war bis zum Übergang auf die Fußarkade etwas kaliberschwächer.

Antibiotisch wurde die Patientin bei mikrobiologischem Nachweis von *Enterobacter cloacae* resistenzgerecht mit Piperacillin-Tazobac abgeschirmt.

Beim geplanten Folgeeingriff imponierte eine gute Granulationstendenz. Die freiliegende Sehne des M. tibialis anterior konnte nach Mobilisierung des umgebenden Gewebes suffizient gedeckt werden.

Die Planung der Defektdeckung erfolgte unter Berücksichtigung der bekannten Vorerkrankungen, des Alters, der fraglichen Compliance der Patientin sowie der Lokalisation des Defekts. Bei freiliegender Achillessehne entschieden wir uns für die Defektdeckung mittels distal gestieltem M.-peronaeus-brevis-Lappen.

**Figure 3:**
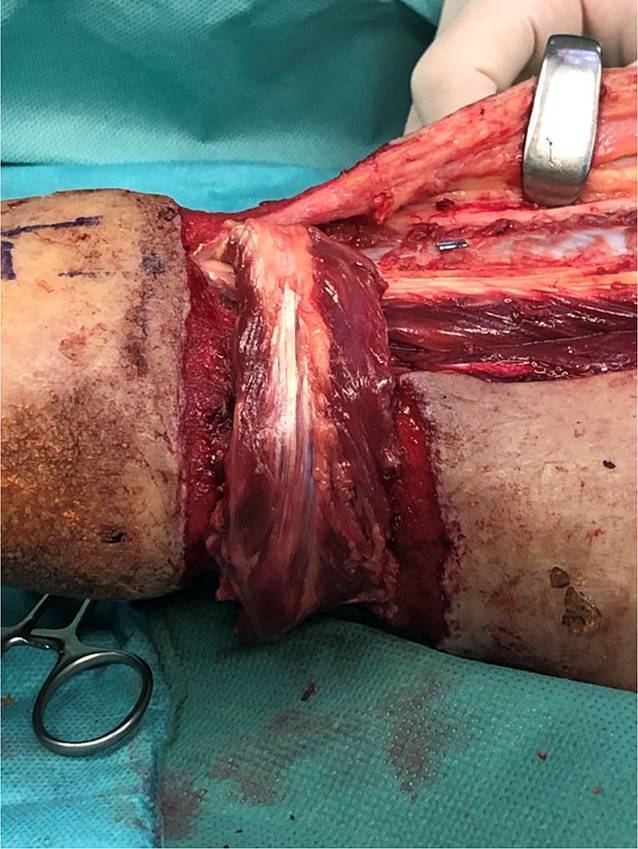

**Figure 4:**
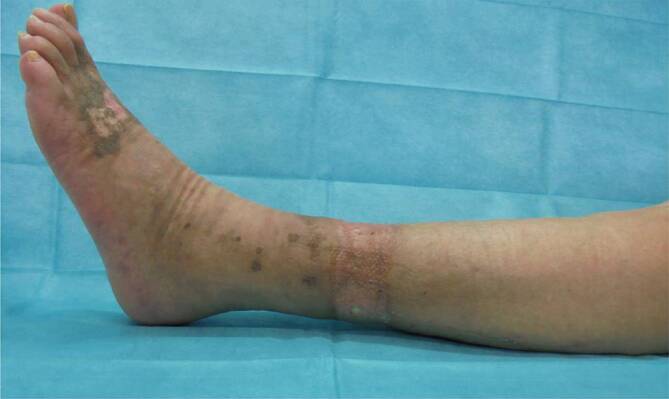

Durch die Skarifizierung und teilweise Entfernung der Muskelaponeurose gelang eine Ausbreitung des M.-peronaeus-brevis-Lappens über die gesamte Wundbreite von 5,5 cm (Abb. 3). Auf den Muskellappen erfolgte die Transplantation von Spalthaut. Der Lappen reichte bis weit nach medial, sodass nur etwa 5 cm des unter der vorgängigen VAC-Therapie gezüchteten Granulationsgewebes anteromedial mit reiner Spalthaut gedeckt werden mussten. Aufgrund der Lokalisation des Lappens an der dorsalen Zirkumferenz und des Ziels einer druckstellenfreien Lagerung wurde anschließend ein externer tibiometatarsaler Rahmenfixateur angelegt und für 14 Tage belassen. Mit dem Lappentraining wurde am 8. postoperativen Tag begonnen. Hierbei kam es nie zu Zeichen einer venösen Stauung. Eine Mobilisation der Patientin unter Belastung des linken Beines wurde ab dem 14. postoperativen Tag begonnen und gestaltete sich problemlos. In den Kontrollen in der Sprechstunde zeigte sich ein vollkommen epithelialisierter Lappen mit einer guten Sprunggelenkbeweglichkeit F/E 30/0/10 (Abb. 4, 5 und 6).

**Figure 5:**
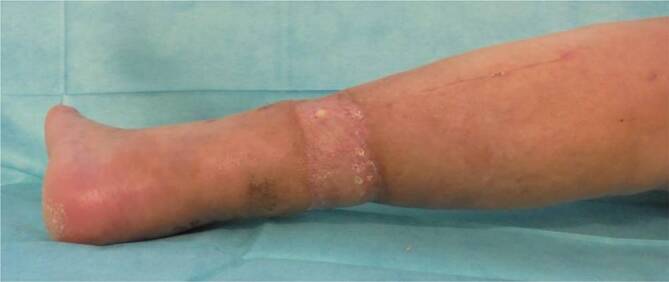

**Figure 6:**
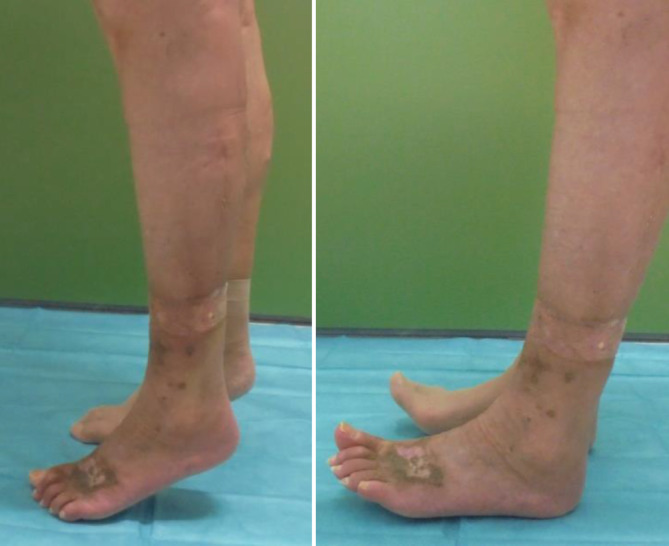

## Diskussion

Dieser Fall zeigt eine seltene Komplikation einer häufig durchgeführten Anwendung zur Prävention thrombembolischer Ereignisse oder venöser Ulzera. Er unterstreicht die Wichtigkeit einer adäquaten Patientenschulung vor Beginn einer Kompressionstherapie mit Berücksichtigung des korrekten Sitzes und regelmäßigen Ausziehens des Kompressionsstrumpfes. In der Fachliteratur gibt es vereinzelt Berichte über Paresen des N. fibularis communis [1], Kompartmentsyndrom [2] und ischämische Nekrosen des Vorfußes [3] durch die unsachgemäße Anwendung der Kompressionstherapie. In einem von Robertson geschilderten Fallbericht kam es durch einen teilweise heruntergerollten Kompressionsstrumpf zu einem Defekt prätibial mit freiliegender M.-tibialis-anterior-Sehne, welche aufgrund von Nekrosen im Verlauf reseziert wurde [4].

Anhand dieses Fallbeispiels wird deutlich, wie wichtig eine Abwägung sämtlicher Alternativen zur Defektdeckung ist. Nur so kann der adäquate individuelle Lappen für den jeweiligen Patienten gefunden werden. Aufgrund des Verletzungsmechanismus und der psychischen Einschränkungen der Patientin fassten wir den Entschluss, keine freie Lappenplastik anzuwenden. Vor dem Hintergrund, dass sich die Patientin bisher alleine in ihrer Wohnung im 1. Obergeschoss versorgt hatte, war der Anspruch, einen Lappen zu wählen, der die Achillessehne suffizient deckt und somit der Patientin die Wiederaufnahme ihres selbstständigen Lebens ermöglicht. Eine Herausforderung in diesem speziellen Fall war der zirkuläre Defekt. Aufgrund der Tatsache, dass der kraniale Rand des Defekts ca. 12,5 cm proximal des Malleolus lateralis lokalisiert war und der Defekt zirkulär 25 cm maß, konnte kein alleiniger Perforatorlappen designt werden, da dieser bis weit in die Poplitea hätte reichen müssen. Möglich wären zwei Perforatorlappen gewesen (aus der A. tibialis posterior und der A. peronaea), hierbei hätten dann jedoch beide Hebedefekte mit Spalthaut gedeckt werden müssen. Eine Defektdeckung mittels Suralislappen war ebenfalls nicht möglich, da der distale oberflächlich verlaufende Stiel entlang der V. saphena parva mit seinem Umkehrpunkt 7 cm proximal des Malleolus lateralis genau im Defektverlauf lag. Die Vorteile der distal gestielten M.-peronaeus-brevis-Lappenplastik lagen auf der Hand:

- schmale Narbe an der Entnahmestelle,
- suffizientes Muskelgewebe zur adäquaten Deckung der Achillessehne und zur Ausplombierung des Defektes,
- Lage des Muskels im tiefen Kompartiment lateral, sodass davon auszugehen war, dass die Blutversorgung dieses Muskels durch die Strangulation des Strumpfes nicht in Mitleidenschaft gezogen wurde,
- geringe Hebestellenmorbidität, da der M. peronaeus longus für die Stabilität des Sprunggelenkes erhalten bleibt,
- der distale Perforator aus der A. fibularis befindet sich meist 5 cm oberhalb der Spitze des Malleolus lateralis und somit im konkreten Fall außerhalb des Haut‑/Weichteildefektes.

Entsprechend der Klassifikation nach Mathes und Nahai wurde der M. peronaeus brevis als Typ III (duale Gefäßversorgung proximal aus der A. tibialis anterior und distal aus der A. fibularis) klassifiziert [5].

Der initial 1971 von Ger beschriebene und von Eren dann im Jahr 2000 für Defekte im distalen Unterschenkel propagierte distal gestielte M.-peronaeus-brevis-Lappen ist relativ einfach und schnell zu heben [6]. Eine Lupenbrillenvergrößerung ist ausreichend, ein Operationsmikroskop nicht erforderlich. Bei dieser asensiblen Lappenplastik bleiben alle 3 Unterschenkelgefäße intakt, sodass sich dieser Lappen auch bei Patienten mit Diabetes mellitus anbietet. Bach konnte zeigen, dass dieser Lappen eine sichere Option für Patientin mit vaskulären Risikofaktoren und höherem Alter ist [7]. Eine Kontraindikation für den Lappen ist ein simultaner Verschluss der A. tibialis posterior und der A. fibularis. Jakubietz et al. empfehlen, besonders bei geriatrischen Patienten, die sich u. U. nicht für einen freien mikrovaskulären Gewebetransfer qualifizieren, als lokale Lappenplastik als letzten Versuch vor der Unterschenkelamputation bevorzugt den M.-peronaeus-brevis-Lappen und Perforatorlappen einzusetzen [8]. In der Literatur ist der erfolgreiche Einsatz dieser Lappenplastik für Defekte an der Ferse, der Achillessehne und am Sprunggelenk beschrieben. Unseres Wissens gibt es jedoch noch keine Fallbeschreibung für einen zirkulären Defekt. Mit seiner Länge von 15–20 cm [9] erwies sich dieser Lappen als ideal, da somit fast die ganze Zirkumferenz gedeckt werden konnte. Durch Muskelatrophie (der motorische Nervenast zum M. peronaeus brevis wird bei der Präparation durchtrennt) und Spalthautkontraktion kommt es im Verlauf der folgenden 6 Monate zu einer Volumenabnahme bis zu 80 % [10]. Es handelt sich hierbei um eine Volumenabnahme des initial sehr kräftigen Muskelbauches und weniger um eine Reduktion der Muskellänge, sodass auch bei der zirkulärer Verwendung keine konstringierenden Effekte des Lappens zu erwarten sind.

## Fazit für die Praxis

Eine Kompressionstherapie ist bei Patienten mit psychischen Begleiterkrankungen mit Bedacht einzusetzen bzw. nur unter engmaschiger Kontrolle durchzuführen. Bei der Wahl des geeigneten Deckungsverfahrens sollte nicht nur die Wunde mit ihren spezifischen Charakteristika berücksichtigt werden, sondern insbesondere auch auf die somatischen und v. a. psychischen Begleiterkrankungen eingegangen werden. Der distal gestielte M.-peronaeus-brevis-Lappen eignet sich auch bei multimorbiden Patienten zur Defektdeckung am Unterschenkel und lässt sich aufgrund seines großen Rotationsradius auch für zirkuläre Defekte sicher anwenden.
